# Extracellular ATP-Induced Alterations in Extracellular H^+^ Fluxes From Cultured Cortical and Hippocampal Astrocytes

**DOI:** 10.3389/fncel.2021.640217

**Published:** 2021-04-30

**Authors:** Ji-in Vivien Choi, Boriana K. Tchernookova, Wasan Kumar, Lech Kiedrowski, Calla Goeke, Marina Guizzetti, John Larson, Matthew A. Kreitzer, Robert Paul Malchow

**Affiliations:** ^1^Department of Biological Sciences, University of Illinois at Chicago, Chicago, IL, United States; ^2^Stritch School of Medicine, Loyola University, Maywood, IL, United States; ^3^Spot Cells LLC, Chicago, IL, United States; ^4^VA Portland Health Care System, Portland, OR, United States; ^5^Department of Behavioral Neuroscience, Oregon Health & Science University, Portland, OR, United States; ^6^Department of Psychiatry, University of Illinois at Chicago, Chicago, IL, United States; ^7^Department of Biology, Indiana Wesleyan University, Marion, IN, United States; ^8^Department Ophthalmology and Visual Sciences, University of Illinois at Chicago, Chicago, IL, United States

**Keywords:** astrocyte, pH, H^+^, ATP, glia, calcium

## Abstract

Small alterations in the level of extracellular H^+^ can profoundly alter neuronal activity throughout the nervous system. In this study, self-referencing H^+^-selective microelectrodes were used to examine extracellular H^+^ fluxes from individual astrocytes. Activation of astrocytes cultured from mouse hippocampus and rat cortex with extracellular ATP produced a pronounced increase in extracellular H^+^ flux. The ATP-elicited increase in H^+^ flux appeared to be independent of bicarbonate transport, as ATP increased H^+^ flux regardless of whether the primary extracellular pH buffer was 26 mM bicarbonate or 1 mM HEPES, and persisted when atmospheric levels of CO_2_ were replaced by oxygen. Adenosine failed to elicit any change in extracellular H^+^ fluxes, and ATP-mediated increases in H^+^ flux were inhibited by the P2 inhibitors suramin and PPADS suggesting direct activation of ATP receptors. Extracellular ATP also induced an intracellular rise in calcium in cultured astrocytes, and ATP-induced rises in both calcium and H^+^ efflux were significantly attenuated when calcium re-loading into the endoplasmic reticulum was inhibited by thapsigargin. Replacement of extracellular sodium with choline did not significantly reduce the size of the ATP-induced increases in H^+^ flux, and the increases in H^+^ flux were not significantly affected by addition of EIPA, suggesting little involvement of Na^+^/H^+^ exchangers in ATP-elicited increases in H^+^ flux. Given the high sensitivity of voltage-sensitive calcium channels on neurons to small changes in levels of free H^+^, we hypothesize that the ATP-mediated extrusion of H^+^ from astrocytes may play a key role in regulating signaling at synapses within the nervous system.

## Introduction

An ever-increasing number of studies suggest strongly that glial cells are far more than the “passive” or “filler” elements originally envisaged years ago when christened “glue” by Rudolf Virchow (Ndubaku and de Bellard, 2008). In addition to their now well-established roles in providing nutrients and scaffolding critical for neuronal growth, proper development, and continued function, glia are now recognized as active participants in the “tripartite synapse,” modulating and regulating signal transmission between neurons and among themselves (Halassa et al., 2007, 2009; Papouin et al., 2017). The removal by glia of neurotransmitter released by neurons is one key mechanism well-known to play an essential role in regulating the extent of neuronal excitation and inhibition (cf. Allen, 2014; Kardos et al., 2017; Malik and Willnow, 2019; Valtcheva and Venance, 2019; Belov Kirdajova et al., 2020 for review). In addition, it has long been suspected that elevations in glial intracellular calcium also lead to modulation of synaptic transfer at synapses, but the precise nature and molecular mechanism(s) of such regulation by glial cells is currently an area of contentious debate (cf. Khakh and McCarthy, 2015; Bazargani and Attwell, 2016; Guerra-Gomes et al., 2017; Fiacco and McCarthy, 2018; Savtchouk and Volterra, 2018; Ashhad and Narayanan, 2019; Kofuji and Araque, 2020; Semyanov et al., 2020).

A number of chemical agents including glutamate, adenosine, serine and GABA have been suggested to act as “gliotransmitters” and act as potential modulators of neuronal activity, although the molecular mechanisms by which these gliotransmitters are released remains controversial (Sahlender et al., 2014; Durkee and Araque, 2019). However, an additional powerful but commonly overlooked mechanism for regulation of synaptic transmission is small changes in levels of extracellular acidity (H^+^). As extracellular acidity increases, protons bind to neuronal calcium channels, reducing the peak calcium influx through these proteins as well as inducing a rightward shift in the transmembrane voltage required to activate these voltage-gated channels (Barnes and Bui, 1991; Barnes et al., 1993). Indeed, in experiments conducted by Stephen Barnes and colleagues using the retina of the tiger salamander, altering extracellular pH to 7.0 completely abolished postsynaptic responses from photoreceptors to second order horizontal cells and was as effective in reducing neurotransmission as 100 μM cadmium, an agent known to potently block calcium influx through voltage-gated calcium channels and to also effectively eliminate all synaptic transmission at this concentration (Barnes et al., 1993). A similar dramatic cessation of neurotransmission from photoreceptors to horizontal cells was reported by Kleinschmidt (1991) upon reducing extracellular pH to 7.2 in the retina of the salamander.

Recent experiments examining radial glial cells isolated from the vertebrate retina (Müller cells) have shown that activation of these glia by extracellular ATP induces a marked increase in extracellular H^+^ flux, acidifying the extracellular milieu (Tchernookova et al., 2018). This ATP-induced extracellular increase in H^+^ flux was detected from Müller cells isolated from a wide range of evolutionarily distant species, ranging from lamprey, skate, tiger salamander, rat, monkey, and human, suggesting a highly evolutionarily conserved response. Further experiments examining the molecular mechanisms responsible for the ATP-elicited H^+^ flux from tiger salamander Müller cells suggested that the response arose from activation of a G-protein coupled ATP receptor and that the H^+^ flux required an increase in calcium released from intracellular stores. It was suggested that this release of H^+^ from Müller cells might play a role in regulating release of neurotransmitter from retinal neurons.

In the present set of experiments, we used H^+^-selective microelectrodes in a self-referencing fashion to show that ATP applied extracellularly to astrocytes cultured from the hippocampus and cortex also elicits a pronounced increase in extracellular H^+^ flux. We also found that the extracellular H^+^ flux is similarly dependent upon an increase in the release of calcium from intracellular stores. We propose that the ATP-mediated increase in extracellular H^+^ flux is likely to be a general property of many types of glia and that this may be a common mechanism by which glial cells modulate neurotransmitter release from neurons throughout the nervous system.

## Materials and Methods

### Preparation of Cell Cultures

All animals were treated in accordance with the protocols approved by the Animal Care Committee (ACC), the Institutional Animal Care and Use Committee (IACUC) of the University of Illinois at Chicago and the federal guidelines listed in the Public Health Service Policy on Humane Care and Use of Laboratory Animals. CD-1 mice pups were purchased from Charles River Laboratories (Wilmington, Massachusetts) and were bred and maintained by the Biological Resources Laboratory (BRL) of the University of Illinois at Chicago. Gestational day 15 pregnant female Sprague-Dawley rats were purchased from Charles River Laboratories (Wilmington, Massachusetts).

Astrocyte cultures were prepared from the hippocampus of postnatal day (PD) 0–1 CD-1 mice or from the cortex of PD 0–1 rats of either sex based on protocols previously described (Guizzetti et al., 1996; Chen et al., 2013). The hippocampi (mice) and cortices (rats) were dissected in 10 ml of HBSS solution (Gibco) containing 10 mM HEPES (pH 7.5), 1 unit/mL penicillin/streptomycin (Invitrogen), and 1 mM of pyruvic acid and cut into smaller pieces and collected into a new tube containing 10 mL of fresh HBSS solution. After two additional washes in 10 mL HBSS solution, hippocampal, or cortical pieces were digested in sterile enzyme solution containing 0.75 mM EDTA, 1.5 mM CaCl_2_, and 25 units/mL of papain for 30 min at 37°C under gentle shaking. The enzymatically digested brain pieces were washed with 5 mL of DMEM (Gibco) containing 1 unit/mL of penicillin/streptomycin (Invitrogen) and 10% fetal bovine serum (complete glial medium) twice, 10 mL of HBSS twice, and again with 5 mL of glial medium twice. About 3 mL of the glial medium from the final wash was discarded and the brain tissues in remaining 2 mL of glial medium were mechanically triturated through a 1 mL pipette. Astrocytes were plated in complete medium at a concentration of 40,000–65,000 cells/mL in 35 mm dishes (Falcon 3001) for H^+^ flux recording and incubated at 37°C in a humified atmosphere of 5% CO_2_- 95% air. The medium was replaced a day after the dissociation, and then every other day after that. In some experiments, cryopreserved cortical astrocyte cultures from Sprague-Dawley rats were purchased from Spot Cells LLC. These cells were thawed and plated using a protocol provided by the manufacturer and cultured as described above for freshly obtained tissue.

For imaging of intracellular calcium alterations, cells were plated on glass coverslips mounted in Petri dish inserts (Spot Cells LLC). Glass coverslips were coated with 50 μg/mL of poly-D-lysine (Sigma) for at least 2 h, rinsed twice with distilled water and air dried before plating cells on them.

#### Staining for GFAP

Astrocytic cultures were fixed with 4% formaldehyde in phosphate buffered saline (PBS) and treated with blocking buffer containing 0.3% Triton X-100 and 3% goat serum in PBS. The cells were then incubated with a primary monoclonal anti-GFAP mouse antibody (1:500, Sigma) and stored in a refrigerator overnight at 4°C. After rinsing with PBS, the cells were incubated with a secondary FITC-labeled anti-rabbit IgG antibody (1:500, Sigma) in blocking buffer for 2 h at room temperature. After washing the cells with PBS, fluorescent staining was analyzed under standard FITC settings (488 nm excitation, 585 nm emission) using digital fluorescence microscopy. Cell dishes not exposed to the primary antibody were used as negative controls.

#### H^+^-Selective Electrode Preparation

The procedure used to prepare H^+^-selective microelectrodes was similar to the protocol described by Smith et al. (1999), Smith and Trimarchi (2001), and Molina et al. (2004). Glass capillary tubes with outer diameter of 1.65 mm and inner diameter of 1.15 mm (King Precision Glass) were pulled to tip diameters of 2–4 μm using a model P-97 Sutter Instruments pipette puller. Pulled pipettes were placed on a metal screen rack tip-side-up; the rack full of pulled pipettes was placed on a glass petri dish, covered with a glass beaker and placed in an oven located in a laboratory safety hood and heated at 200°C for at least 24 h for drying. The micropipettes were then silanized with the vapor produced by adding a 0.1 mL drop of N, N-dimethyltrimethylsilyalamine (Sigma) onto the bottom of the glass Petri dish. After 30 min, the beaker covering the pipettes was rotated 180 degrees to allow vapor to escape into the air hood, and the silanized micropipettes were taken out of the oven and set aside to cool down for several minutes. Cooled pipettes were stored in a glass desiccator with desiccant to prevent moisture formation inside. Silanized micropipettes were then back-filled with 100 mM KCl (pH 7.4) buffered with 10 mM HEPES, and a positive pressure applied through a syringe filled with air to fill the tip all the way to the end of the micropipette with fluid. Using an inverted microscope, the tip of the pipette was then placed in contact with a silanized glass pipette of greater diameter containing the highly selective H^+^ resin, hydrogen ionophore 1-cocktail B (Fluka), and about 30 μm of H^+^ ionophore was drawn into the smaller silanized pipette tip. H^+^-selective microelectrodes were calibrated with standard pH 6.0, 7.0, and 8.0 solutions (Fisher Scientific). Only microelectrodes with Nernstian voltage slopes of 45–60 mV/per pH unit were used in experiments. Control experiments were also conducted to ensure that drugs at the concentrations applied did not alter the sensitivity of the H^+^ sensors.

#### Solutions

For most H^+^ flux experiments, solutions contained 1 mM of the pH buffer HEPES along with 140 mM of NaCl, 5 mM KCl, 2.5 mM CaCl_2_, 2 mM MgCl_2_, and 15 mM glucose and were adjusted to pH 7.4 using NaOH. In some experiments, bicarbonate buffer solutions containing 124 mM NaCl, 4 mM KCl, 1.25 mM KH_2_PO_4_, 1.25 mM CaCl_2_, 1.5 mM MgSO_4_, 26 mM NaHCO_3_, and 10 mM glucose were used; this was bubbled with 95% O_2_ and 5% CO_2_ to achieve a pH of 7.4. Recordings were made in a cell dish containing 4 mL of solution, and during each solution exchange, 20 mL of the next solution was exchanged to ensure near complete washout of previous solution, followed by a short period of time to allow the solutions to once again become mechanically quiet and to allow the H^+^ gradient to be restored. The self-referencing technique relies on the presence of an H^+^ concentration gradient between the two points being measured (see below). Continual superfusion of solutions over the cell would eliminate this concentration gradient. A home-built superfusion chamber, described fully in Kreitzer et al. (2017), was employed to exchange solutions and apply pharmaceutical agents; this same chamber allowed maintenance of the 95% O_2_/5% CO_2_ mix used in experiments with bicarbonate as the primary extracellular pH buffer, and also allowed the maintenance of a 100% oxygen atmosphere in experiments designed to eliminate potential contributions from CO_2_ in the normal room air.

#### H^+^ Flux Recordings

To make self-referencing recordings from isolated cells, the culture medium in a dish containing cells was first replaced by a recording saline and placed on the stage of a Zeiss 40CFL inverted microscope resting on an air isolation table and enclosed in a Faraday cage to reduce electrical interference. A ground electrode made from a capillary tube filled with 3% agar and 3 M NaCl was placed in the dish and connected to the head stage of the self-referencing amplifier via a sintered silver/silver chloride electrode (WPI). The electronics, software, and mechanical control of electrode movement were the same as described in Molina et al. (2004) and Kreitzer et al. (2017) and were products of the BioCurrents Research Center, Woods Hole MA.

For self-referencing measurements of extracellular H^+^ flux, a H^+^-selective microelectrode was first positioned about 1–2 μm away from an isolated cell, a voltage was recorded, and the electrode then moved to a second position 30 μm away and another reading taken at this background location. An H^+^-dependent differential voltage signal was then obtained by subtracting the voltage signal at the distant position from the position adjacent to the cell membrane. This differential measurement is at the heart of self-referencing, and removes much of the inherent slow electrical drift that is present in virtually all ion-selective electrodes. An important assumption of the technique is that the frequency of movement of the microelectrode between the two points (0.3 Hz in these studies) is fast enough so that the electrical drift is virtually identical at the two points, but not fast enough to significantly stir the solution and disturb the diffusional H^+^ gradient being measured from the cell. This differential measurement combined with signal averaging is estimated to increase the sensitivity of the ion-selective microelectrodes by 1,000 times (Somieski and Nagel, 2001). As noted in prior publications (Smith and Trimarchi, 2001; Molina et al., 2004; Kreitzer et al., 2007, 2017), the signals generated by the self-referencing H^+^-selective microelectrodes used in these experimental conditions are not likely to arise from surface potentials or other stray sources of extracellular voltages. For example, extracellular voltage fields generated by isolated cells are usually in the nanovolt range and below the sensitivity of ion-selective self-referencing probes (Kuhtreiber and Jaffe, 1990; Smith et al., 1999), and electrical potentials from local boundary conditions associated with membrane surface charges (McLaughlin et al., 1971, 1981) drop with the Debye length and do not extend into the medium by more than tens of angstroms (cf. Cevc, 1990); our H^+^-selective microelectrodes were located at least 1 μm away from the surface of cells. Finally, at the end of every recording, control background differential signals were measured at a position 600 μm away from the cell in the Z-plane; at this location, the levels of H^+^ should be the same at the two positions of electrode movement, resulting in a differential signal near zero. Recordings from which the differential signal differed by more than 25 μV from the expected zero value were discarded.

#### Calcium Imaging

Intracellular [Ca^2+^] was assessed using the Ca^2+^-sensitive fluorescent indicator Fura-2 AM (Grynkiewicz et al., 1985). Astrocytes growing in Spot Cells' Petri dish inserts were loaded with Fura-2, by filling the inserts with 50 μl of 4 μM Fura-2AM in cell culture medium and incubating (37°C, 5% CO2) for 10 min. The inserts were then transferred to chambers that fit a microscope stage (Kiedrowski and Feinerman, 2018), where they were superfused with 1 mM HEPES saline buffer solution to remove the extracellular Fura-2 AM. Intracellular Fura-2 fluorescence imaging was carried out using a digital fluorescence imaging system with DIC optics. The system included a 20× Zeiss Fluar 20×, NA 0.75 objective, a Zeiss AxioObserver D1 microscope (Zeiss, Göttingen, Germany), and excitation/emission filter wheels (Sutter Instrument, Novato CA) controlled by MetaFluor 7.7.8 software (Molecular Devices LLC, Sunnyvale, CA, USA). Agents of interest were applied onto the cells via superfusion that was conducted using a MPRE-8 manifold and 8-channel valve switch, cFlow8 (Cell MicroControls, Norfolk, VA, USA). Images of fluorescence (4 × 4 binning) emitted at 510 nm and excited at 340 and 380 nm were taken every 10 s for off line analysis. The F340/F380 ratio was measured in regions of interest (ROIs) and used as an indicator of intracellular [Ca^2+^]. At the end of each experiment, cells were exposed to 10 μM of ionomycin and 3 μM of FCCP for saturation of Fura-2 with Ca^2+^. For data analysis, background fluorescence measured in cell-free regions was subtracted from raw fluorescence values at F340 and F380 measured in the ROIs.

#### Data Analysis

For most self-referencing isolated cell recordings, differential voltage values quickly reached new plateau levels, and the mean value of the last 90 points were used in data analysis. In some cases (e.g., in response to application of glutamate), cellular responses to a solution exchange were rather transient. For these cases, the first 20 points out of 100 were used in data analysis. The Wilcoxon signed-rank test was used to determine the level of statistical significance in experiments for which the N was 5. For all other experiments, mean ± standard error of the mean (SEM) and *P*-values were calculated using Prism software (GraphPad), and Student's paired *t*-tests were used to determine statistical significance. In histograms, a single asterisk indicates *P*-values between 0.01 and 0.05, two asterisks indicate *P* < 0.01.

## Results

**Figure 1:**
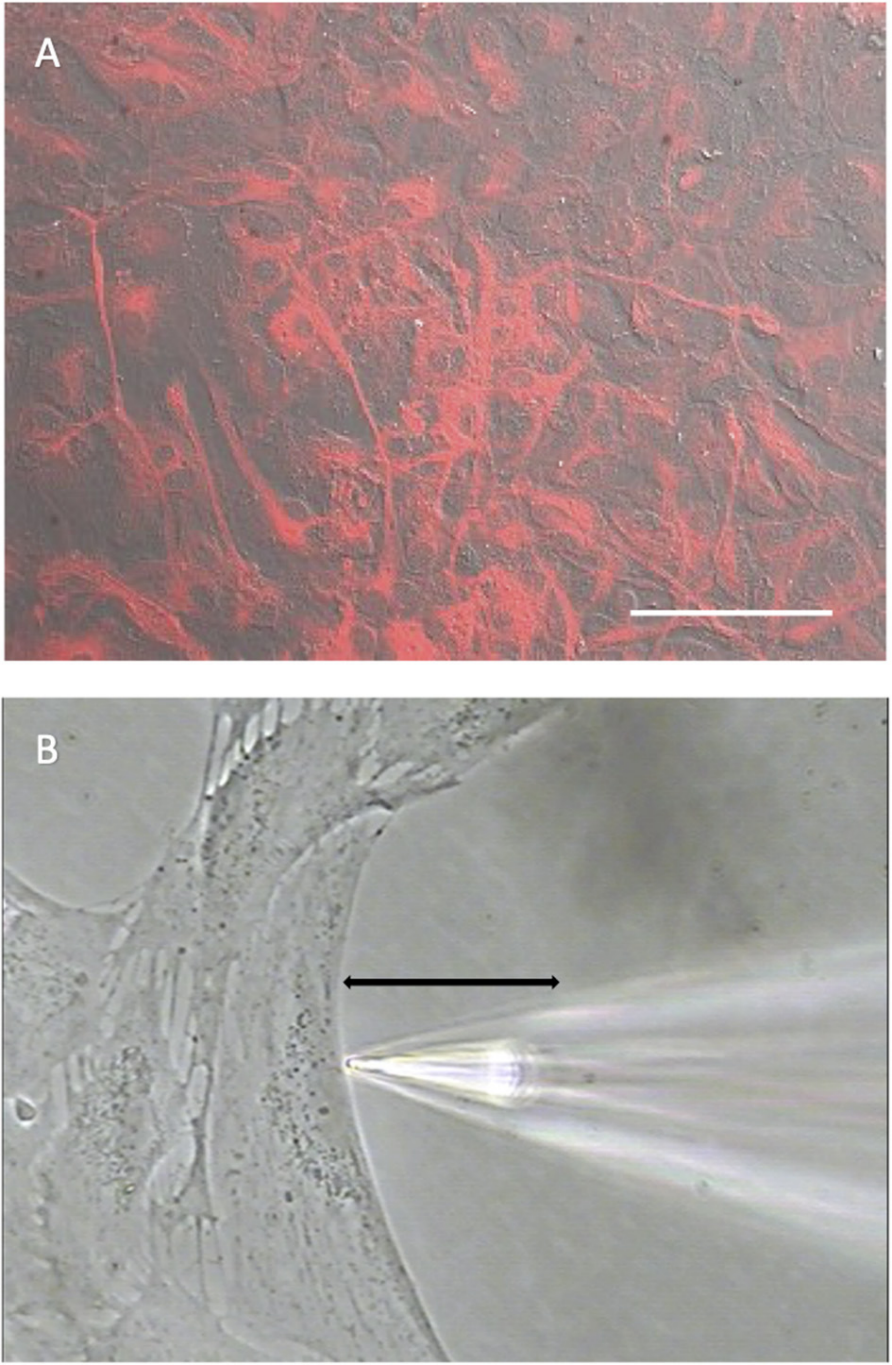
Cells cultured from mouse hippocampus display robust staining for the glial cell marker GFAP. **(A)** Low power photomicrograph combining DIC image with fluorescent staining for GFAP in a confluent dish of cells cultured for 11 days from mouse hippocampus. Red indicates signal from GFAP. The signal was not observed in negative controls (not shown). White scale bar indicates 100 μm. **(B)** High power photomicrograph depicting positioning of an H^+^ selective microelectrode adjacent to a cell cultured from mouse hippocampus. Black line indicates the 30 μm lateral movement the H^+^ selective electrode would travel from the near pole adjacent to the plasma membrane (pictured) to a background location 30 μm away.

Experiments were carried out on primary cell cultures prepared from rat cortex and mouse hippocampus using culturing conditions designed to limit the growth of neurons and microglia. To confirm that the cells in culture had characteristics of astrocytes, cell cultures were immune-stained with an antibody specific for glial fibrillary acidic protein (GFAP), an intermediate filament protein typically expressed at high concentrations in astrocytes (Dixon et al., 2004; Sofroniew and Vinters, 2010; Zhang et al., 2017). Figure 1A shows a photomicrograph illustrating the extensive and intense staining for GFAP detected in cells prepared from mouse hippocampus in a dish with a nearly confluent cell culture, with almost all cells observed displayed high levels of GFAP.

Figure 1B shows a photomicrograph depicting the typical recording arrangement used to measure H^+^ fluxes from single cells. Recordings were made in dishes plated at a lower density of cells than shown in Figure 1A. Under such conditions, cells tended to be large and flat with extensive broad extensions, a morphology typical of cells also identified as astrocytes in previous studies (Fawthrop and Evans, 1987; Schildge et al., 2013). Plating at a lower density ensured that there was ample open area free of cells for at least several hundred microns away from the edges of a cell identified for recording, a key requirement for the self-referencing recordings used here to examining extracellular H^+^ fluxes. Once a cell with appropriate morphology and ample spacing had been identified, an H^+^-selective microelectrode was positioned ~1–2 μm from the edge of the membrane of the cell as well as about 1–2 μm from the bottom of the dish, as shown in Figure 1B. An initial reading was first obtained from the H^+^-selective microelectrode at this position; the electrode was then translated laterally to a background reference location 30 μm away into the open area devoid of cells, and a second reading from the H^+^-selective microelectrode taken (for further details associated with data collection, please see methods). The reading obtained at the background location was then subtracted from that obtained at the edge of the cell, resulting in a signal that reflects an extracellular H^+^ flux. A key advantage of this self-referencing approach is that it effectively eliminates the slow electrical drift inherent in all ion-selective electrodes, which can otherwise be large enough to significantly obscure measurements of H^+^ efflux from single cells (Smith and Trimarchi, 2001).

A characteristic feature of astrocytes is the presence of H^+^-dependent transporters for the neurotransmitter glutamate, and activation of these transport proteins leads to an extracellular alkalinization (Rose and Ransom, 1996; Rose et al., 2017). We therefore initially examined alterations in H^+^ flux from cells cultured from mouse hippocampus that were bathed in a Ringer's solution containing 1 mM HEPES and then challenged with 500 μM glutamate. Prior to stimulation, a standing differential signal of 129 ± 22 μV was detected from seven cells, indicating that the solution adjacent to the plasma membrane of the cell was more acidic than the point 30 μm away. Following a control application of a bolus of the same Ringer's solution bathing the cell, the standing H^+^ flux was virtually unchanged at 123 ± 21 μV (*P* = 0.32). Upon application of 500 μM glutamate, the differential H^+^ signal declined to 69 ± 22 μV, indicating a significant drop in the level of acidity adjacent to the membrane as compared to the originally detected standing H^+^ flux (*P* = 0.004), consistent with what would be expected from the transport of glutamate into the cell.

**Figure 2:**
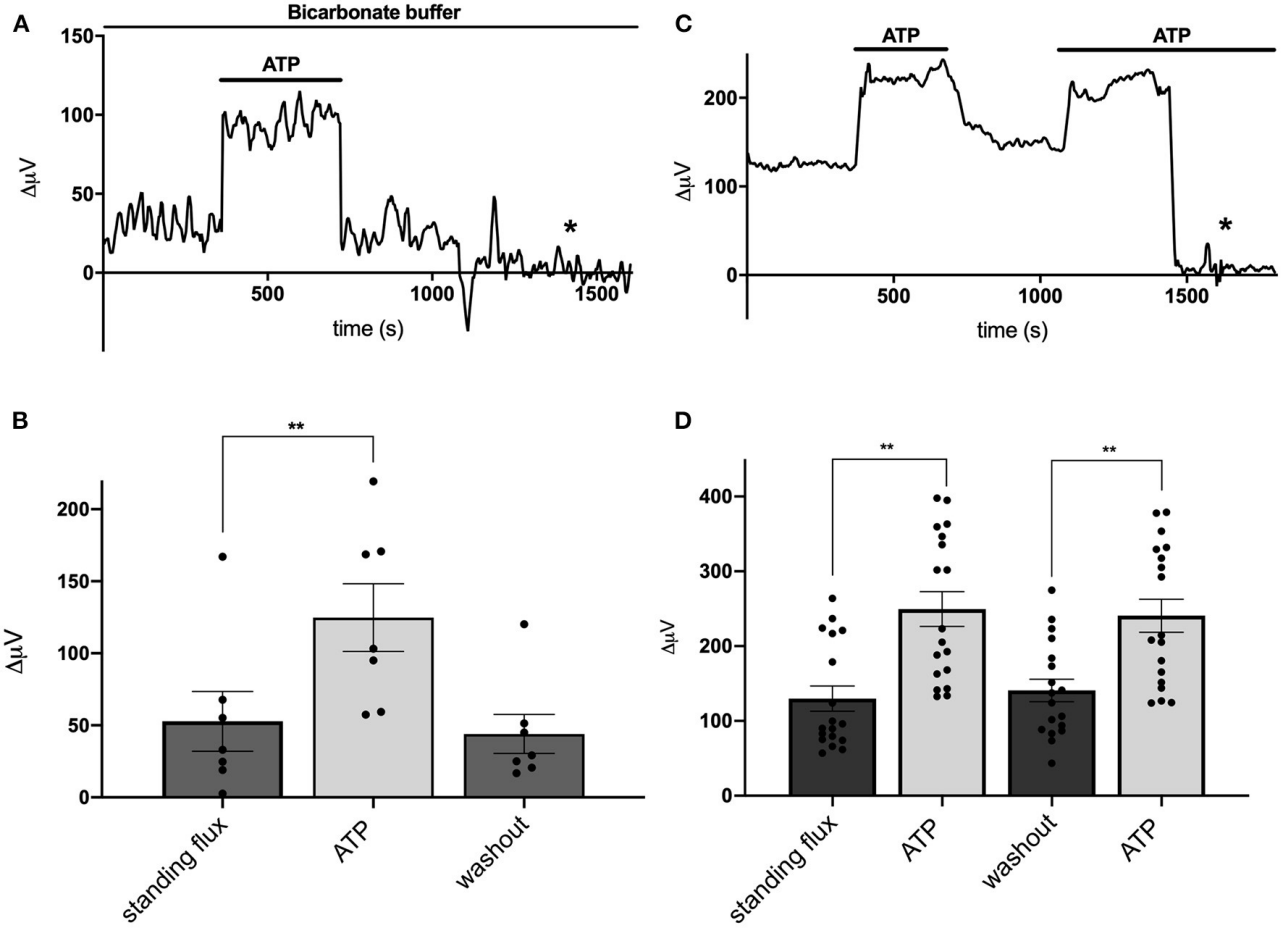
Responses of cells identified as astrocytes cultured from mouse hippocampus to applications of 100 μM ATP. **(A)** Response from one cell bathed in saline buffered with 26 mM bicarbonate to 100 μM ATP. A standing H^+^ flux of about 30 μV was observed prior to stimulation. Addition of 100 μM ATP to the bath solution induced a detectable increase in H^+^ flux. Upon removal of ATP, the H^+^ flux signal returned to its pre-stimulus baseline. From about 1,250 s until the end of the trace (indicated by the asterisk), the electrode was moved to a position 600 μm above the cell, a location where the differential response should be close to 0 μV. **(B)** Average H^+^ flux from seven cells bathed in 26 mM bicarbonate saline before, during and after the application of 100 μM ATP; *N* = 7. **(C)** Response from one cell bathed in saline containing 1 mM HEPES as the primary extracellular pH buffer to applications of 100 μM ATP. Prior to challenge with ATP, the cell displayed a standing H^+^ flux signal of abut 120 μV. Addition of 100 μM ATP induced an increase in the H^+^ flux signal. Removal of ATP resulted in the return of the H^+^ flux signal near to its original pre stimulus level. A second application of 100 μM ATP again led to a marked increase in H^+^ flux. At about 1,450 s, the electrode was repositioned 600 μm above the cell (designated by the asterisk); at this background location, sampling between two points 30 μm apart gave a differential signal close to 0 μV. **(D)** Average H^+^ flux signals from cells bathed in saline containing 1 mM HEPES as the primary extracellular pH buffer; *N* = 18. **indicates *P* < 0.01.

We next examined the effects of the addition of extracellular ATP on H^+^ flux from cells cultured from mouse hippocampus. Figure 2A shows a response from one cell typical of those obtained when recordings were made with cells bathed in a solution containing 26 mM bicarbonate, the primary buffer for extracellular pH under normal physiological conditions. The initial portion of the trace shows a small standing differential signal which we refer to as the standing H^+^ flux prior to stimulation. Upon exchange of the normal bicarbonate Ringer's solution with one containing 100 μM ATP, a significant increase in the signal associated with H^+^ flux was detected. Replacement of the ATP containing solution with the normal bicarbonate Ringer's solution lacking ATP resulted in an H^+^ flux similar in magnitude to the standing H^+^ flux initially detected. At about 1,250 s into the recording, the electrode was elevated to a position 600 μm above the cell and a control set of differential measurements was made until the end of the recording (marked by the asterisk in this and in other recordings). With the electrode at this control location, the concentration of H^+^ in the solution should be identical at the two locations of electrode movement. The output of the H^+^-selective electrode should thus be the same at the two locations of electrode movement and the differential signal should be close to zero, which is what was observed. This control was done in all recordings obtained in the present work.

Figure 2B shows the quantitative results obtained for H^+^ flux from seven cells cultured from mouse hippocampus and bathed in the Ringer's solution in which the primary buffer was 26 mM bicarbonate. Under these conditions, cells had an average standing H^+^ flux prior to stimulation of 53 ± 21 μV. Challenging the cells with 100 μM ATP led to an increase in total H^+^ flux to 125 ± 25 μV, a significant increase in the overall differential signal detected (*P* = 0.0004). Upon restoration of normal Ringer's solution lacking ATP, the flux decreased to 44 ± 14 μV, a value statistically indistinguishable from the initial standing flux (*P* = 0.40). Extracellular ATP induced similar increases in extracellular H^+^ flux from cells cultured from rat cortex possessing astroglial-like characteristics. 100 μM ATP significantly increased the size of the H^+^ signal from a standing value of 43 ± 18 μV to 186 ± 39 μV in six cells examined (*P* = 0.004).

When stimulated by extracellular ATP, radial glial cells of the retina, known as Müller cells, demonstrate a significantly increased H^+^ efflux that is not dependent on the presence of extracellular bicarbonate or bicarbonate transport mechanisms. To test whether the ATP-induced response from cells cultured from hippocampus was similarly independent of extracellular bicarbonate, recordings from cells cultured from mouse hippocampus were obtained using a solution in which 1 mM HEPES was used as the primary extracellular pH buffer and to which no bicarbonate was added. Figure 2C shows a sample trace from one cell recorded under these conditions. A standing H^+^ flux was again detected prior to stimulation. Application of 100 μM ATP to the extracellular solution induced a notable increase in the overall differential H^+^ flux. Removal of ATP brought the flux back to near the original standing H^+^ flux, and a second stimulation with 100 μM ATP again increased the size of the detected signal from the H^+^-selective microelectrode. The later portion of the trace again reflects a control recording with the electrode placed at 600 μm above the cell resulting in a differential H^+^ signal close to zero. Figure 2D shows the averaged signals obtained from 18 cells and reveals a standing H^+^ flux from unstimulated cells and a significant and repeatable increase in H^+^ flux upon addition of 100 μM ATP (*P* < 0.0001). Removal of ATP again led to return of the H^+^ flux signal to levels similar to the initial standing H^+^ flux. A second application of ATP again increased the H^+^ flux signal detected (*P* < 0.0001). The size of this second increase was statistically indistinguishable from the initial response to ATP (*P* = 0.14).

**Figure 3:**
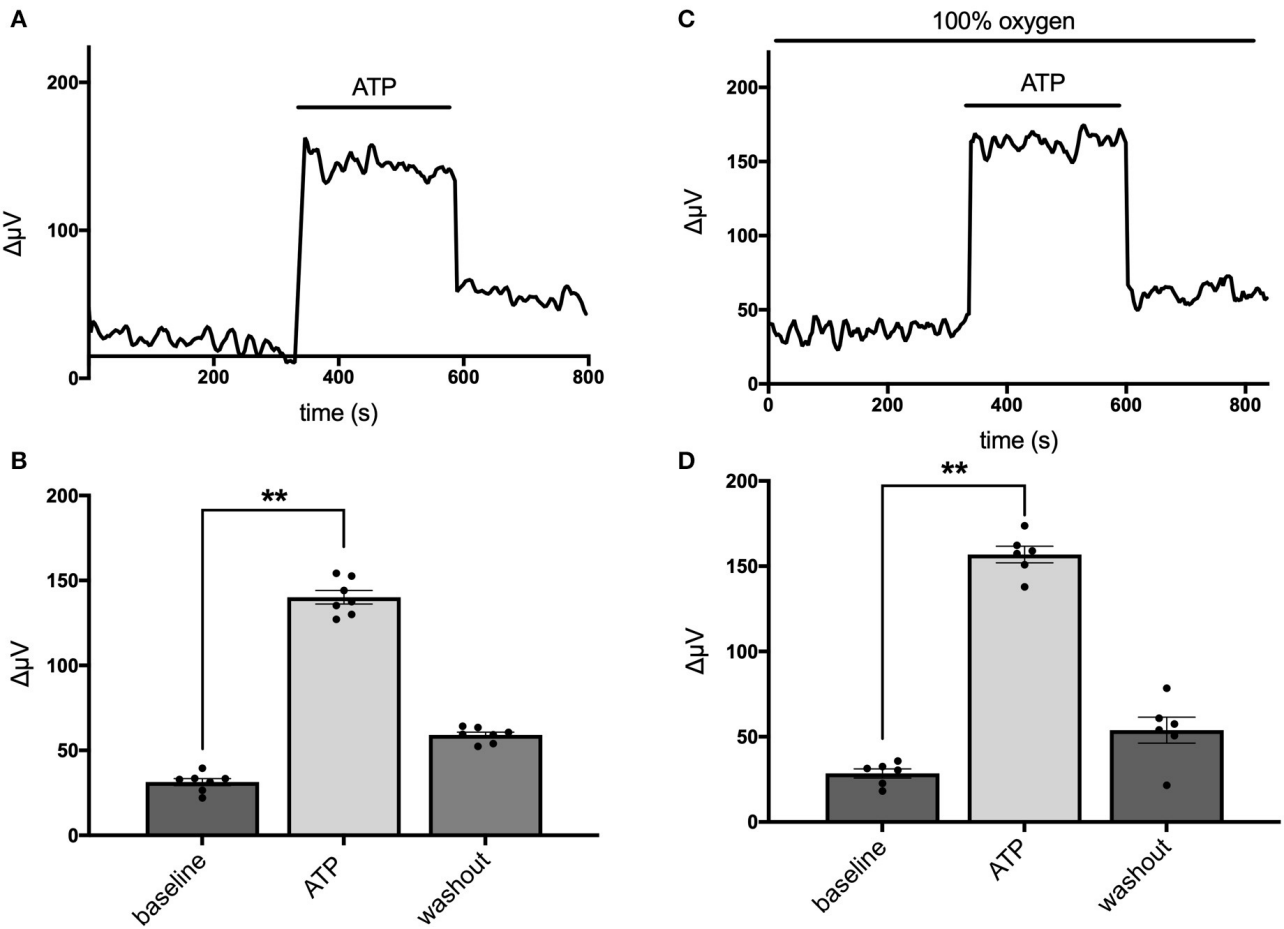
Responses of cells from cryopreserved mouse hippocampal cultures to 100 μM ATP bathed in a saline solution containing 1 mM HEPES and saturated either with normal air or 100% oxygen. **(A)** Trace from one cell maintained in normal air in standing saline followed by application and then removal of 100 μM ATP. **(B)** Quantitative data averaged from cells in normal air; *N* = 6. **(C)** Trace of H^+^ flux signal from one cell maintained in 100% oxygen. **(D)** Quantitative data of H^+^ signals obtained from cells in saline containing 100% oxygen; *N* = 7. **indicates *P* < 0.01.

Astrocytes have been shown to possess a sodium-dependent bicarbonate transporter with particularly high affinity, such that the low levels of bicarbonate resulting from CO_2_ in the normal atmosphere could potentially provide sufficient substrate for this transporter to function (Theparambil et al., 2014, 2017; Theparambil and Deitmer, 2015). Activation of this transporter could also lead to an alteration in detected levels of H^+^ adjacent to the membrane of a cell even in the absence of added bicarbonate. To test whether this mechanism might account for H^+^ fluxes initiated by extracellular ATP, we recorded from mouse hippocampal astrocytes immersed in solutions containing 1 mM of the pH buffer HEPES that had been bubbled continuously with 100% oxygen for at least 15 min, to drive off possible contributions from CO_2_ in the air. Figure 3 shows individual responses and averaged data from cells recorded in normal air and a separate population of cells bathed in 100% oxygen. Figure 3A depicts the standing H^+^ flux signal, response to 100 μM ATP, and return of the H^+^ flux signal upon removal of ATP from a single cell maintained in normal air. Figure 3B shows quantitative data averaged from six cells maintained in normal air. In this cell population, the average H^+^ flux prior to stimulation was 31 ± 2 μV and addition of 100 μM ATP increased the overall H^+^ flux signal to 140 ± 4 μV (*P* < 0.0001). Figure 3C shows a recording from one cell maintained in 100% oxygen demonstrating that 100 μM ATP induced a clear increase in H^+^ flux in cells in this condition. Figure 3D shows results from a separate population of cells recorded in solutions with 100% oxygen. In seven cells examined in Ringer's solution containing 100% oxygen, 100 μM ATP increased the H^+^ signal from a standing H^+^ flux of 29 ± 3 μV to 157 ± 5 μV (*P* < 0.0001). Thus, ATP elicited H^+^ fluxes from cells recorded in oxygen and no atmospheric CO_2_ were actually marginally larger in magnitude compared to cells maintained in normal air by about 17% (*P* = 0.02).

**Figure 4:**
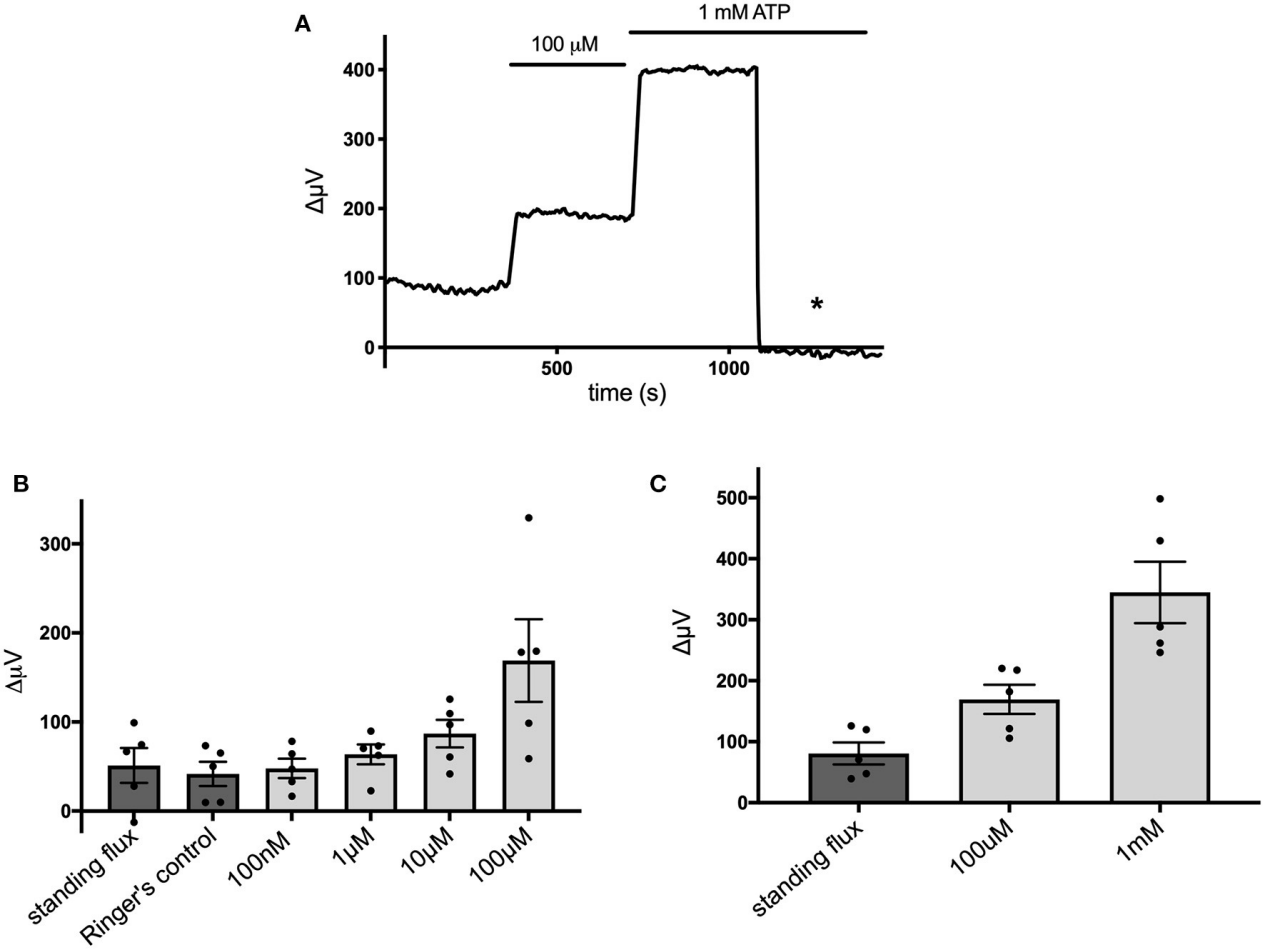
Responses of cells cultured from hippocampus to different concentrations of extracellular ATP. **(A)** Response of one cell in saline solution containing 1 mM HEPES to application of 100 μM and 1 mM ATP. **(B)** Average responses of cells to concentrations of extracellular ATP ranging from 100 nM to 100 μM; *N* = 5. **(C)** Average responses of additional cells to 100 μM and 1 mM extracellular ATP; *N* = 5.

Figure 4 shows that the ATP-elicited extracellular H^+^ fluxes from cells cultured from mouse hippocampus were dependent upon the dose of extracellular ATP applied. Figure 4A shows a sample trace from a cell first exposed to 100 μM ATP and then when challenged with 1 mM ATP. Figure 4B shows the average response from five cells to applications of extracellular ATP as concentrations were raised from 100 nM up to 100 μM, and Figure 4C shows averaged results from an additional set of five cells when challenged with 100 μM followed by 1 mM ATP.

**Figure 5:**
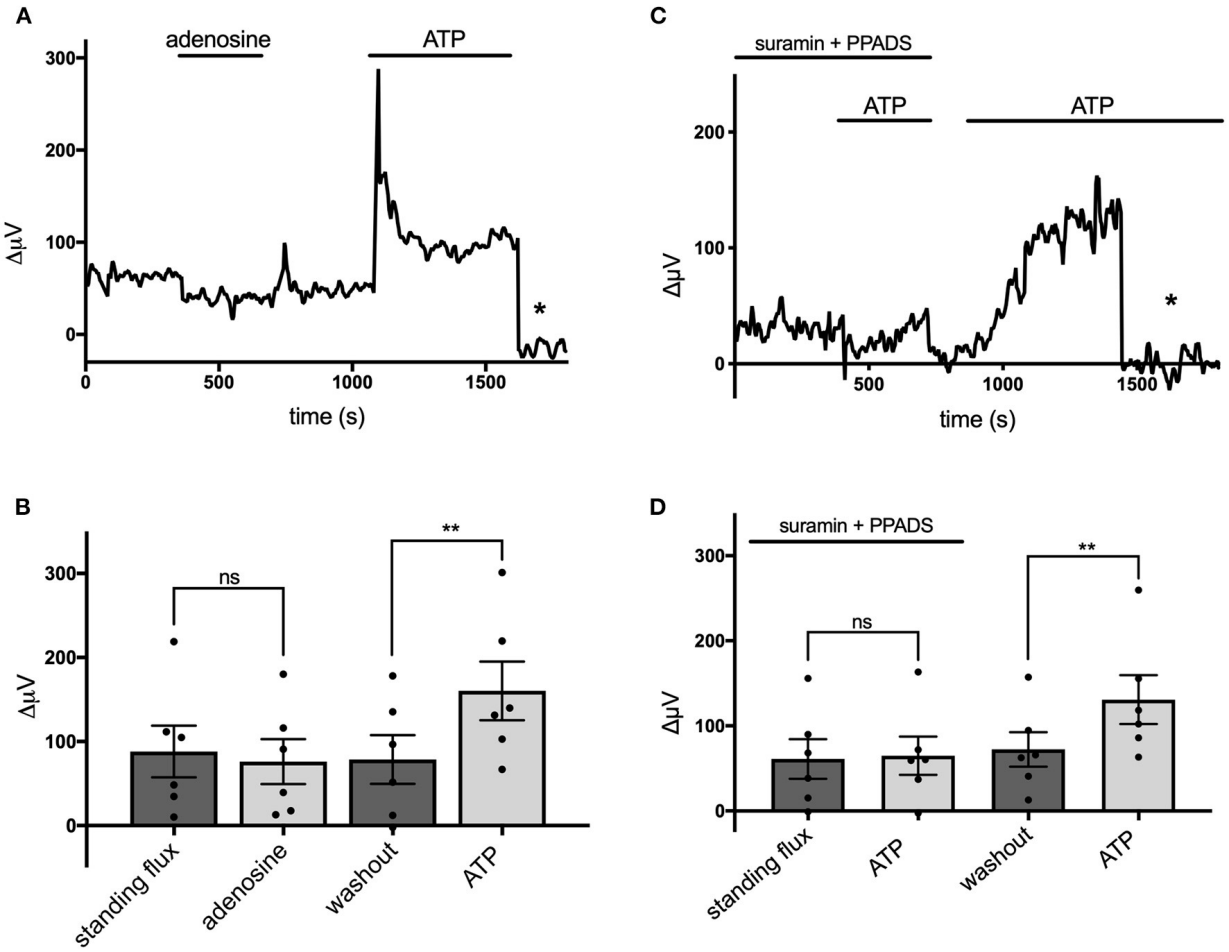
Adenosine does not alter H^+^ flux signals while suramin and PPADS reduce H^+^ flux. **(A)** Response of a single cell cultured from mouse hippocampus to 100 μM adenosine and 100 μM ATP. Asterisk indicates signal obtained when the H^+^-selective electrode had been moved to a control position 600 μm above the cell. **(B)** Quantitative results to the applications of 100 μM adenosine and 100 μM ATP from cells (*N* = 6). **(C)** Response of a single cell to 100 μM ATP first in the presence of 500 μM suramin and 500 μM PPADS and then again to 100 μM ATP following removal of suramin and PPADS. Asterisk again indicates recordings of the H^+^-selective electrode when at a control position 600 μm above the cell. **(D)** Quantitative results averaged from cells to 100 μM ATP in the presence of 500 μM suramin and 500 μM PPADS and response to 100 μM ATP following removal of suramin and PPADS; *N* = 6. **indicates *P* < 0.01; ns indicates not significantly different (*P* > 0.05).

Extracellular ATP is known to be broken down to adenosine by the action of ecto-ATPase enzymes, and activation of adenosine receptors is known to be a potent modulator of the activity of many cells in the nervous system (Wilson and Mustafa, 2009). We therefore tested the effects of adenosine on cells cultured from mouse hippocampus and identified as astroglial in nature. Figure 5A shows a trace from a single cell that was unresponsive to application of 100 μM adenosine but showed a notable increase in H^+^ flux when challenged by 100 μM ATP. Figure 5B shows averaged results from six cells. In this population, the average H^+^ flux in the presence of 100 μM adenosine was 76 ± 27 μV, statistically indistinguishable from the standing H^+^ signal of 88 ± 31 μV observed prior to challenge with adenosine. In these same cells, 100 μM ATP increased the H^+^ flux signal to 160 ± 35 μV, a statistically significant increase compared to the standing flux (*P* = 0.002). While adenosine was ineffective in inducing an increase in H^+^ flux, chemicals known to block ATP receptors were effective in significantly reducing the H^+^ flux induced by ATP. Figure 5C shows a trace from one cell challenged with 100 μM ATP with the cells bathed in a solution containing 500 μM suramin and 500 μM PPADS, agents known for their ability to block P2 ATP-activated receptors. The mean response from six cells to 100 μM ATP in the presence of PPADS and suramin was 65 ± 23 μV, which was not significantly different from the standing flux (61 ± 23 μV) observed in the presence of those blockers (*P* = 0.68). Following washout of PPADS and suramin, 100 μM ATP increased the H^+^ flux signal from a new standing flux value of 72 ± 20 μV to 131 ± 29 μV (shown graphically in Figure 5D). A similar block of ATP-elicited increases in H^+^ flux by suramin was observed in cells cultured from rat cortex. The mean H^+^ flux signal detected in response to application of 50 μM ATP in the presence of 1 mM suramin was 33 ± 4 μV in five such cells, which was not significantly different from the standing flux of 24 ± 9 μV observed in the presence of the blocker alone (*P* = 0.19). Following washout of both suramin and ATP, these cells displayed a standing H^+^ flux signal of 39 ± 5 μV, and application of 50 μM ATP then increased the size of the H^+^ flux signal to 159 ± 29 μV (*P* = 0.006).

**Figure 6:**
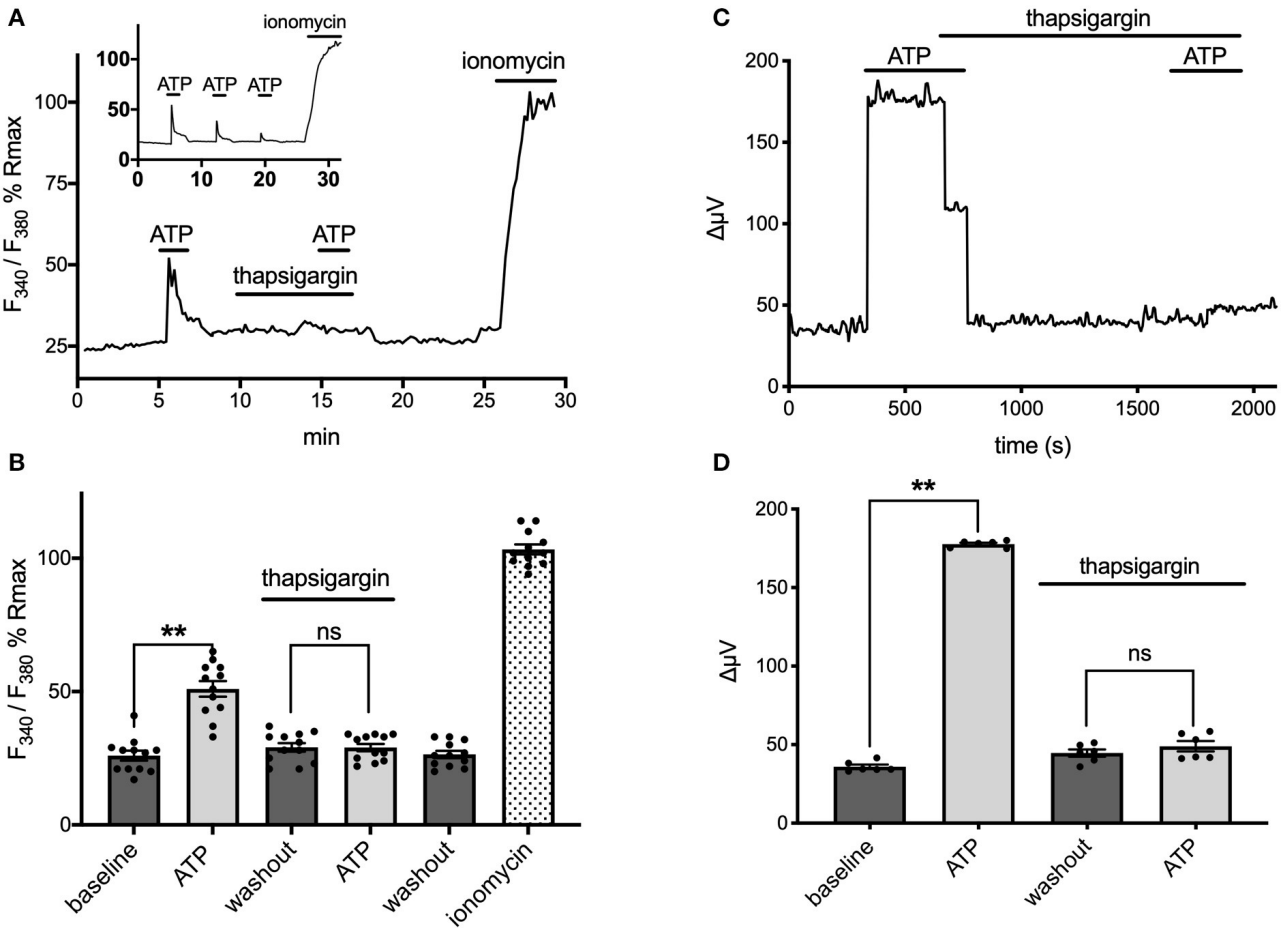
ATP-induced alterations in H^+^ flux require increases in intracellular calcium. **(A)** Measurements of changes in intracellular calcium from cells cultured from mouse hippocampus as reported by the calcium indicator fura-2. Trace shows averaged percentage change in the ratio of Fura-2 fluorescence from 12 astrocytes imaged simultaneously plotted as a percentage of the maximal change in ratio observed upon addition of the calcium ionophore ionomysin. Challenge of the cells with 100 μM ATP resulted in an increase in the ratio of Fura-2 fluorescence indicative of an increase in intracellular calcium. Following washout of the ATP and return of the signal to baseline, cells were superfused with 1 μM thapsigargin for several minutes and then challenged a second time with 100 μM ATP, which now induced little change in the Fura-2 ratio. The thapsigargin and ATP were then removed from the dish; after several additional minutes, the calcium ionophore ionomycin was added to determine a maximal change in fluorescent ratio. Inset shows typical response to multiple applications of ATP under control conditions with no thapsigargin present. **(B)** Data from the same cells presented in histogram format, again as a percentage of the maximal induced change in ratio induced by application of ionomycin; *N* = 12. **(C)** Self-referencing trace from a single cell cultured from cryopreserved mouse hippocampal cells showing alteration in H^+^ flux to 100 μM ATP. The cell was then bathed in 1 μM thapsigargin, ATP washed off, and the cell left to sit in thapsigargin for several minutes. A second application of 100 μM ATP now produced little change in the H^+^ flux signal. **(D)** Effects of thapsigargin on H^+^ flux signal; *N* = 6. **indicates *P* < 0.01; ns indicates not significantly different (*P* > 0.05).

Extracellular ATP has been shown to increase intracellular calcium in astrocytes via activation of G-protein-linked ATP receptors that result in the release of calcium from intracellular stores (cf. Agulhon et al., 2008; Wang et al., 2009; Bazargani and Attwell, 2016; Guerra-Gomes et al., 2017 for review). We therefore tested whether the ATP-initiated H^+^ flux detected with self-referencing H^+^ selective electrodes similarly required increases of intracellular calcium. Ratiometric measurements of fluorescence changes in cells loaded with Fura-2AM and excited at 340 and 380 nm were used to examine alterations in intracellular calcium and plotted as a percentage of the saturating fluorescence change induced by addition of the calcium ionophore ionomycin. Figure 6A shows the change in the fluorescent ratio with time averaged from 12 astrocytes cultured from mouse hippocampus and monitored simultaneously during one such experiment. Following challenge with 100 μM ATP, a significant increase in the ratio of 340/380 induced fluorescence was detected, indicating a significant elevation of intracellular calcium as expected; the standing fluorescence ratio prior to stimulation was 26 ± 2% of the maximal signal, compared to a ratio of 51 ± 3% in the presence of 100 μM ATP (*P* < 0.001) (shown graphically in Figure 6B). ATP thus produced an ~100% increase in the fluorescent ratio. ATP was then washed off, and 1 μM thapsigargin, an agent known to prevent re-accumulation of calcium into intracellular stores, was added for several minutes. Cells were then challenged again with 100 μM ATP, and as can be seen in the figure, ATP now failed to elicit a significant rise in intracellular calcium. The standing fluorescence ratio in the presence of 1 μM thapsigargin was 29 ± 2%; upon addition of 100 μM ATP, the fluorescence ratio remained at 29 ± 1% (*P* = 0.84). In contrast, in control experiments conducted in the absence of thapsigargin, a second application of 100 μM ATP produced an alteration in the fluorescent ratio that was 71% the size of the initial application of ATP (*N* = 11); the inset to Figure 6A shows one example of a control experiment in which ATP was applied multiple times. Thus, thapsigargin was able to significantly depress the rise in intracellular calcium normally initiated by ATP.

The same concentration of thapsigargin that effectively eliminated ATP-induced increases in intracellular calcium also potently inhibited ATP-induced increases in H^+^ flux. Figure 6C shows the H^+^ flux signal obtained from one astrocyte cultured from cryopreserved hippocampal cells. Application of 100 μM ATP produced a clear increase in H^+^ flux. 1 μM thapsigargin was then added to the bath and ATP removed several seconds after, and the H^+^ flux signal returned to its pre-stimulus level. Challenge of the cell with 100 μM ATP several minutes later with thapsigargin still present now failed to induce a large increase in the H^+^ flux signal. Figure 6D shows averaged responses from six cells cultured from cryopreserved mouse hippocampal cells. 100 μM ATP increased the H^+^ flux signal from a pre-stimulus level of 36 ± 1 μV to 178 ± 1 μV (*P* < 0.0001). In the presence of 1 μM thapsigargin, 100 μM ATP failed to induce a significant increase in the same set of cells. After several minutes in thapsigargin, the standing H^+^ flux signal from these cells was at 45 ± 2 μV; addition of 100 μM ATP in the continued presence of thapsigargin now resulted in an H^+^ flux signal of 49 ± 3 μV, not significantly different from the pre-stimulus standing flux detected in the presence of thapsigargin alone (*P* = 0.081).

**Figure 7:**
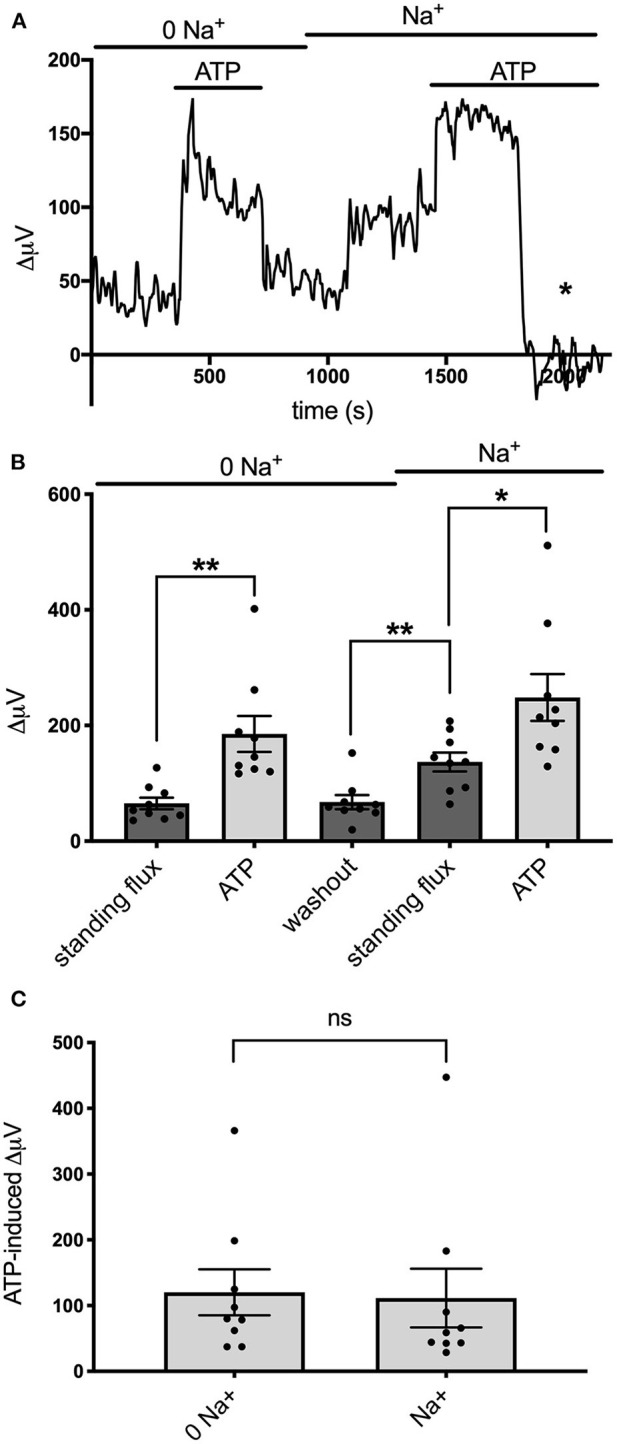
Effects of alterations in extracellular sodium on ATP-induced increases in H^+^ flux. **(A)** Trace showing H^+^ flux signals in response to 100 μM ATP from a single cell cultured from mouse hippocampus first when bathed in 0 mM extracellular sodium and then when extracellular sodium was restored to normal levels. Restoration of sodium induced a notable increase in the standing H^+^ flux. Asterisk indicates response of self-referencing H^+^-selective electrode when in a control location 600 μm above the cell. **(B)** Quantitative results averaged from cultured mouse hippocampal cells showing H^+^ flux in cells first bathed in 0 mM sodium and then when normal levels of sodium were restored; *N* = 9. *indicates 0.01 < *P* < 0.05; **indicates *P* < 0.01. **(C)** Responses to ATP from the same cells when the contribution of the standing H^+^ flux observed prior to application of ATP was subtracted out; *N* = 9. ns, indicates not significantly different (*P* > 0.05).

Recent work examining the molecular mechanisms underlying ATP-induced H^+^ extrusion from retinal Müller glia suggests that the majority of acid is provided by Na^+^/H^+^ exchange activity (Tchernookova et al., 2021). Consequently, we sought to determine if this was also the case for the ATP-induced extrusion of H^+^ from brain astrocytes. To explore this question, we examined H^+^ fluxes under conditions in which extracellular sodium was removed and replaced with an equivalent amount of choline, a large cation that cannot substitute for sodium in Na^+^/H^+^ exchange. Figure 7A shows the result of this ionic substitution protocol on the response from one astrocyte cultured from mouse hippocampus. With the cell bathed in 0 mM extracellular sodium (all sodium replaced by equimolar choline), 100 μM ATP still produced a sizable increase in extracellular H^+^ flux as measured with a self-referencing H^+^-selective electrode and washing out the ATP brought the H^+^ flux signal back near to its original size. Restoration of extracellular sodium to normal levels resulted in an increase in the standing H^+^ flux signal, and addition of 100 μM ATP produced a further increase in H^+^ flux. Panel B of Figure 7 shows quantitative results averaged from nine such cells. With cells bathed in 0 mM sodium, addition of 100 μM ATP increased the H^+^ flux signal from an initial standing flux value of 65 ± 10 μV to 185 ± 31 μV, a statistically significant increase in the size of the flux (*P* = 0.009). Washing out the ATP with the cell still in 0 mM sodium brought the signal back close to its initial level (68 ± 12 μV). Replacement of the 0 sodium/choline solution with normal extracellular sodium Ringer's solution increased the standing H^+^ flux signal to 137 ± 16 μV, significantly larger than the standing flux observed in 0 sodium Ringer's solution (*P* = 0.0003). Addition of 100 μM ATP to the same cells further increased the H^+^ flux signal to 249 ± 41 μV, a significant increase compared to the standing H^+^ flux observed upon restoration of normal extracellular sodium (*P* = 0.037). In order to determine the effect of sodium specifically on ATP-induced increases in H^+^, the size of the ATP-induced signals were compared after having subtracted out the value for the standing H^+^ flux. This is shown in Figure 7C and plots the average amplitude of the ATP-induced H^+^ flux signal in the same nine cells after having subtracted out the value for the standing flux prior to ATP application. The increase in H^+^ flux due to addition of 100 μM ATP was 120 ± 35 μV with cells bathed in 0 mM sodium and 112 ± 35 μV in normal sodium solution, values that were not statistically different from one another (*P* = 0.57) and arguing against a large role for Na^+^/H^+^ exchange in mediating ATP-elicited increases in H^+^ flux.

**Figure 8:**
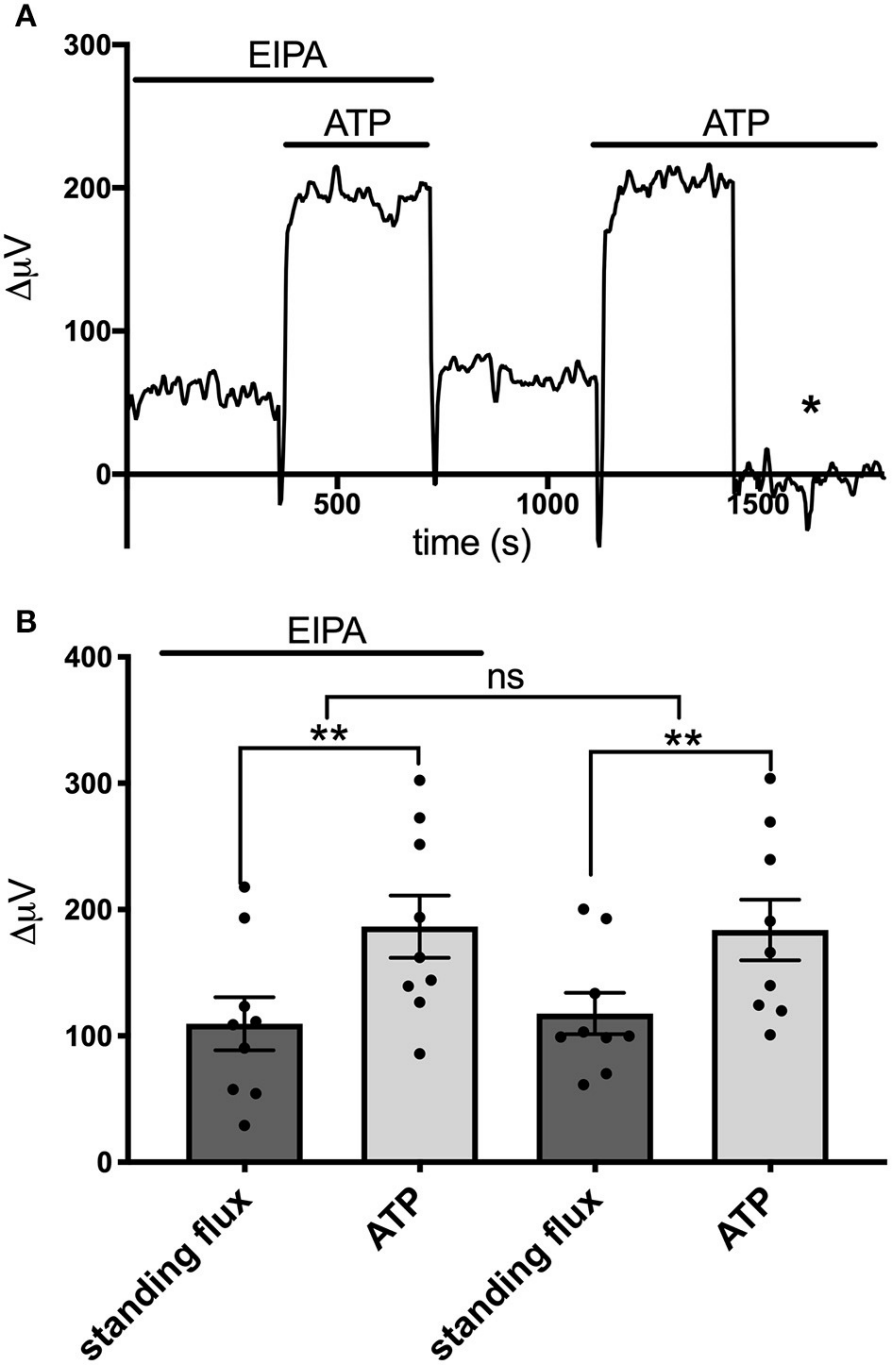
Lack of effect of the Na^+^/H^+^ inhibitor EIPA on H^+^ flux. **(A)** Trace showing H^+^ flux signal from a single astrocyte cultured from mouse hippocampus upon application of 100 μM ATP first in the presence of 200 μM EIPA and then again after the EIPA had been removed from the bath. **(B)** H^+^ flux signals averaged from cells cultured from mouse hippocampus in the presence of EIPA and following washout of the drug; *N* = 9. **indicates *P* < 0.01; ns indicates not significantly different (*P* > 0.05).

We also examined the effects of EIPA, a potent pharmacological inhibitor of Na^+^/H^+^ exchange, on the ATP-induced increases in H^+^ flux. EIPA has been reported to have an IC50 of ~0.02–2.5 μM in inhibiting the activity of isoforms NHE1, NHE2, NHE3, and NHE5 (Attaphitaya et al., 1999; Masereel et al., 2003). Figure 8A shows the response from one astrocyte cultured from mouse hippocampus to applications of 100 μM ATP in the presence and then absence of 200 μM EIPA. An obvious increase in the size of the H^+^ flux upon addition of 100 μM ATP was apparent in both conditions. Figure 8B shows the size of the H^+^ flux signals averaged from nine such cells. 100 μM ATP increased the H^+^ flux from a standing value of 10 ± 21μV to 187 ± 25 μV (*P* = 0.0009) in the presence of EIPA. Following washout of the EIPA, 100 μM ATP increased the H^+^ flux from a standing value of 118 ± 16 μV to 182 ± 24 μV (*P* = 0.002). The size of the ATP-induced responses in the presence and absence of EIPA were not statistically significantly different (*P* = 0.75).

## Discussion

The data presented here demonstrate that extracellular ATP applied to cells cultured from mouse hippocampus and rat cortex possessing characteristics typical of astrocytes induces an increase in the flux of acid (H^+^) from the cells. Extracellular ATP also induces an increase in levels of intracellular calcium, and this calcium appears to be required for the increase in H^+^ flux, since abolishment of ATP-induced rises in intracellular calcium using thapsigargin also eliminate the increase in H^+^ flux measured with self-referencing H^+^ selective electrodes.

H^+^ fluxes monitored from mouse hippocampal and rat cortical astrocytes using self-referencing electrodes have a number of features that are similar to H^+^ fluxes previously examined from isolated retinal radial glia commonly referred to as Müller cells using similar self-referencing techniques (Tchernookova et al., 2018). Brain-derived astrocytes and retinal radial glia both display small standing H^+^ fluxes prior to activation that are likely associated with the removal of internal H^+^ generated by normal metabolism, and ATP applied extracellularly promotes a sizable increase in H^+^ efflux from both cell types. The increase in H^+^ flux induced by extracellular ATP likely results from activation of metabotropic P2 ATP receptors known to be present on both sets of cells, as judged by the ability of the response to be significantly reduced by the ATP receptor blockers suramin and PPADS. H^+^ flux responses from both cultured astrocytes and isolated retinal radial glia are not altered by application of adenosine, and the ATP-elicited increase in H^+^ flux from both sets of cells require a rise in intracellular calcium as judged by the ability of thapsigargin to eliminate both ATP-initiated increases in intracellular calcium and ATP-induced alterations in H^+^ flux. ATP-induced increases in H^+^ flux are also detected from both cultured astrocytes and Müller cells regardless of whether the primary extracellular pH buffer is provided by physiologically relevant concentrations of bicarbonate or 1 mM HEPES (Tchernookova et al., 2018).

Both astrocytes and Müller cells extend fine processes that wrap and envelop nearby synaptic complexes established by neurons (Wang et al., 2017; cf. Vasile et al., 2017; Zhou et al., 2019). Both sets of cells are thus in an ideal position to be able to detect chemical signals released by neurons at synapses and to modulate release of neurotransmitter at these crucial neuronal connections. Given the high sensitivity of voltage-gated calcium channels to small changes in extracellular acidity (Barnes et al., 1993; Tombaugh and Somjen, 1996; Shah et al., 2001; Doering and McRory, 2007; Saegusa et al., 2011; Han et al., 2017), we propose that the ATP-initiated increase in H^+^ efflux from both astrocytes and Müller cells may well be a key mechanism regulating neurotransmitter release by neurons, acting as an inhibitory feedback loop to reduce release of neurotransmitter. Vesicles in neurons containing neurotransmitter are also known to contain ATP and release that ATP upon fusion with the plasma membrane (cf. Zimmermann, 1994; Pankratov et al., 2006; Abbracchio et al., 2009; Burnstock and Verkhratsky, 2012 for review). We propose that the fusion of neuronal vesicles with the plasma membrane results in the release of both neurotransmitter and ATP, and that the released ATP activates ATP metabotropic receptors on the glia. This would result in an increase in intracellular calcium released from intracellular stores in glia, which ultimately results in the increased extrusion of H^+^ from the glia. According to this hypothesis, H^+^ released by glia would bind to calcium channels on neurons, reducing the peak amount of calcium entering through the channel as well as shifting the voltage-dependent opening of the channels to more depolarized values. The resulting decrease in the influx of calcium into the neurons would result in a decrease in the calcium-dependent fusion of vesicles to the plasma membrane and a consequent decrease in neurotransmitter release by the neurons.

A second important impact likely to result from the extrusion of acid from glial cells is enhanced removal of neurotransmitter from the extracellular milieu by H^+^-dependent transporters known to be present in glia and neurons (Grewer and Rauen, 2005; Soto et al., 2018). For example, hippocampal astrocytes are known to possess transport proteins for the key neurotransmitter glutamate that act by ferrying three sodium ions and one proton along with glutamate into the cell while also exporting one potassium ion (Nicholls and Attwell, 1990; Owe et al., 2006; Vandenberg and Ryan, 2013; Rose et al., 2017). The removal of neuronally-released glutamate by uptake into astrocytes thus leads to an extracellular alkalinization, and is indeed what we and others have detected from cultured astrocytes upon application of glutamate. The co-release of ATP with glutamate from neuronal vesicles would activate H^+^ extrusion from the glia into the extracellular fluid, a process that would supply much needed substrate for the further removal of glutamate from the synaptic cleft. In a careful examination of flux coupling of the EAAT3 glutamate transporter, Zerangue and Kavanaugh (1996) reported an affinity constant for H^+^ of 26 nM during glutamate transport, corresponding to a pH of about 7.58. This value implies that small changes of extracellular H^+^ around normal physiological levels of pH could indeed significantly impact the transport of glutamate into cells. The expected enhancement of the removal of neurotransmitter via addition of H^+^ extruded by astrocytes, coupled with the reduction in the release of neurotransmitter due to H^+^-mediated decrease of calcium into neurons, would both result in inhibiting the flow of neuronal information transfer at the synapse.

Studies examining the three dimensional pattern of calcium dynamics in both awake animals and brain slices suggest that calcium increases in individual astrocytes are scattered throughout the cell, highly compartmentalized within predominantly local regions, and heterogeneously distributed regionally and locally (Bindocci et al., 2017; Savtchouk et al., 2018). Moreover, these same studies also indicate that astrocytes can respond locally to quite minimal axonal firing with time-correlated changes in intracellular calcium. These data suggest that potential regulation of neurotransmitter release by H^+^ into the extracellular fluid could be highly localized, and that extrusion of H^+^ initiated by local increases in calcium may modulate synaptic strength at a synapse-by-synapse scale. The thousands of fine processes from astrocytes wrapping various separate synapses could thus potentially act independently from one another, with ATP released at just that synapse acting to locally influence synaptic strength by highly localized release of H^+^.

ATP can also be released from the glial cells themselves, a phenomenon that has been implicated in the production of waves of intracellular calcium across many glia (Arcuino et al., 2002; Coco et al., 2003; Koizumi et al., 2003; Anderson et al., 2004; Scemes and Giaume, 2006; Bowser and Khakh, 2007; Halassa et al., 2007). Release of ATP from glial cells has also been shown to have effects on synaptic signaling and neuronal activity (Newman, 2003, 2015; Illes et al., 2019). While the mechanism by which glial cells release ATP remains a matter of considerable contention, a number of studies have provided evidence that the release is dependent upon a rise in intracellular calcium. We suggest that H^+^ efflux from glial cells mediated via waves of ATP across multiple glia may represent a key component underlying the phenomenon known as “spreading depression,” characterized as a slow wave of depression of electrical activity observed in many areas of the nervous system (Kunkler and Kraig, 1998; Torrente et al., 2014; Cozzolino et al., 2018; Wu et al., 2018). We hypothesize that the associated depression of electrical activity results from the release of H^+^ from the glial cells, acting on the neuronal calcium channels to reduce the amount of neurotransmitter released.

The ATP-elicited H^+^ fluxes from cultured astrocytes did not appear to be highly dependent upon extracellular sodium. That observation and the fact that fluxes were not significantly reduced in solutions designed to reduce contributions of CO_2_ from the air (by bubbling solutions with 100% oxygen) make it highly likely that the ATP-induced H^+^ fluxes we report here are not due to the activity of a high affinity sodium-dependent bicarbonate transporter. The ATP-initiated increases in H^+^ flux were also not significantly reduced by EIPA, an agent known to be a potent inhibitor of Na^+^/H^+^ exchange activity. The lack of dependence on extracellular sodium and the inability of Na^+^/H^+^ inhibitors to reduce the flux differ from the ATP-induced alteration in H^+^ flux previously detected in the radial glial cells (Müller cells) isolated from the retina of the tiger salamander. Upon removal of extracellular sodium, ATP-initiated H^+^ fluxes from Müller cells were reduced by about 70%, and ATP-initiated H^+^ fluxes were also inhibited by several pharmacological agents known to interfere with Na^+^/H^+^ exchange (Tchernookova et al., 2021). These differences suggest that the molecular mechanism mediating the majority of H^+^ flux in astrocytes cells is likely to be different from that in the retinal radial glial cells. One attractive potential alternative candidate remaining is the activity of monocarboxylate transporters, which are thought to play an important role in enabling astrocytes to provide lactate to neurons for their energy needs and which require co-transport of lactate with H^+^ (Pierre and Pellerin, 2005; Jha and Morrison, 2018, 2020). The release of lactate would likely be greatest when neuronal activity is highest, which would coincide with the highest levels of extracellular ATP co-released with neurotransmitter when numerous vesicles fuse. Future studies will be required to determine the particular protein(s) mediating H^+^ efflux as well as other steps involved in the ATP-initiated signal transduction cascade.

The hydrolysis of ATP to ADP and an additional phosphate group by ecto-ATPases has been suggested to lead to an acidification of the extracellular solution within synapses of the nervous system (Vroman et al., 2014). However, the extracellular acidifications measured here are unlikely to be caused by such a mechanism. The ATP-induced increase in H^+^ flux was virtually abolished by 1 μM thapsigargin, a compound that exerts its effects by blocking the reuptake of intracellular calcium into internal stores, and by the addition of the P2 receptor blockers suramin and PPADS. Neither of these agents is known to have any effect on ecto-ATPases. If the ATP-elicited increase in extracellular H^+^ flux was due to the acidifying actions of an ecto-ATPase, then our H^+^-selective self-referencing probes should still have detected an increase in ATP-induced extracellular H^+^ flux when either thapsigargin or the P2 receptor blocking agents were added to the bath.

The amplitude of the ATP-induced H^+^ flux signal detected from cells in 1 mM HEPES was similar to that obtained with cells bathed in 26 mM HCO_3_. While the overall bulk buffering capacity of the 26 mM bicarbonate solution is expected to be greater than the solution containing 1 mM HEPES, the kinetics of the bicarbonate buffering system in the absence of carbonic anhydrase is also relatively slow and can be rate-limiting, with interconversion between CO_2_ and HCO_3_ taking 30 s or more to reach equilibrium (Maren, 1988; Spitzer et al., 2002). The combination of the slow kinetics of bicarbonate buffering coupled with the very close positioning of the H^+^-selective microelectrode (~ 1 μm) to the source of H^+^ extrusion from the plasma membrane makes it likely that relatively few protons interact with bicarbonate ions to produce CO_2_ prior to being detected by the H^+^-selective sensor. In an unbuffered solution the diffusion coefficient for H^+^ has been reported to be 9.3 ^*^ 10^−5^ cm^2^^*^ sec^−1^, and even within the cytoplasm of a cell containing a mixture of immobile buffering agents along with mobile bicarbonate ions, the diffusion coefficient of H^+^ has been estimated to be 1.4–2.1 ^*^ 10^−6^ cm^2^^*^ sec^−1^ (Al-Baldawi and Abercrombie, 1992; Spitzer et al., 2002). In the latter condition, the time it takes for a proton to travel 1 μm (the distance of the H^+^-selective sensor used in these experiments from the plasma membrane of the cell) is about 2 ms (Al-Baldawi and Abercrombie, 1992). Thus, because of the limited degree of H^+^ buffering expected to take place over this short time and distance, it is perhaps not surprising that the size of the H^+^ flux signals from cells in solutions buffered with bicarbonate or HEPES are likely to be similar in overall magnitude.

A previous study examining acid efflux from astrocytes cultured from the neopallium of 1 day old rats using microphysiometry, an entirely different technique (McConnell et al., 1992), also suggests that extracellular ATP induces acid efflux (Dixon et al., 2004). In that study, it was noted that cariporide, an inhibitor of Na^+^/H^+^ exchange, suppressed the initial portion of ATP-induced H^+^ efflux, and the authors argued for a role for Na^+^/H^+^ transport in this process. However, the 5 μM cariporide used in that study depressed the ATP-elicited H^+^ efflux measured by microphysiometry by a maximum of only about 30%, indicating that the majority of acid release was not due to extrusion by NHE1 exchange. One challenge with the interpretation of microphysiometry results is the requirement in those experiments for responses from very large numbers of cells. While an indication of the exact number of cells used in each microphysiometry experiment was not listed, with techniques employed at the time, on the order of 10^6^ cells or more were needed to obtain reliable signals with this technique (McConnell et al., 1992). The authors note that ~99% of cultured cells in their cultures expressed abundant GFAP and had morphological characteristics similar to those expected to astrocytes, but also note that the cultures used did have small numbers of cells with other characteristics. Given the nature of the microphysiometry experiments (measuring the sum of acid released by all the cells present), it is possible that some portion of the measured H^+^ fluxes may have come from cells other than astrocytes. One major advantage of the self-referencing H^+^ microelectrode method used here is the ability to restrict measurements to alterations in extracellular H^+^ fluxes from single cells possessing a clear astrocytic morphology. On the other hand, the high spatial resolution of H^+^-selective microelectrodes also means that we might have consistently positioned the sensors at regions of the cell low in Na^+^/H^+^ activity and could have missed higher levels in other locations around the cell. Another difference in methodology is that the experiments conducted with microphysiometry were done on confluent cell cultures grown for 11 days, while in the present set of experiments cells were examined at earlier times prior to confluence, and it may well be that the physiological properties of the astrocytes are different in the two sets of culturing conditions. One feature that both methods have in common, however, is the difficulty of attempting to estimate what the actual change in extracellular H^+^ concentration might be under normal physiological conditions where astrocytes wrap and envelop neuronal synapses. As noted by Dixon et al. (2004), extrapolation of results from cell cultures to the brain requires consideration of the buffering properties and geometric characteristics of the intact tissue, including the distance of astrocytic process from the synapse and the location and density of the transporters exporting H^+^ from the astrocytes.

Given the very high sensitivity of neuronal transmission in response to quite small changes in extracellular H^+^, and the observations demonstrating H^+^ efflux from astrocytes when activated by ATP in the present work, experiments to directly measure changes at the level of synapses in the intact brain mediated by activation of astrocytes are clearly called for. A number of important issues remain to be addressed by such future studies. For example, a recent study has suggested that activation of glial P2Y receptors results in the release of bicarbonate from astrocytes (Theparambil et al., 2020) and has been proposed as a mechanism to moderate alterations in H^+^ in the nervous system. The production of H^+^ and HCO_3_ are intimately related, with both resulting from the interaction of CO_2_ with water that is greatly facilitated by the enzyme carbonic anhydrase, and many glial cells have been reported to have high concentrations of this enzyme (cf. Cammer and Tansey, 1988; Nagelhus, 2005; Theparambil et al., 2017, 2020). It seems likely, then, that activation of glial P2Y receptors leads to the production and release of both H^+^ and HCO_3_. The overall impact of these two chemical species on synaptic activity will depend upon the specific location of H^+^ and HCO_3_ transporters as well as on the level of extracellular carbonic anhydrase present within the synaptic cavity. Our previous studies on H^+^ efflux from retinal radial glial (Müller) cells suggest release of protons from areas likely to be associated with synaptic connections, while sodium coupled HCO_3_ transporters on these same cells are much more abundantly expressed in the basal end foot of the cell close to the vitreous humor, suggesting potential differential impacts from the spatial inhomogeneity of release of H^+^ and HCO_3_ produced within the cell (Newman, 1996; Kreitzer et al., 2017; Tchernookova et al., 2018). Also, as noted earlier, the uncatalyzed conversion of HCO_3_ to CO_2_ is quite slow. Even if H^+^ and HCO_3_ were released at the same subcellular location, the effects of H^+^ could be swift, significant, and relatively prolonged if little carbonic anhydrase were present in the synaptic cavity. The impact of concomitantly released HCO_3_ on the time course of extracellular acidification could be dramatically altered if significant concentrations of carbonic anhydrase, which can speed the conversion reaction by a factor of ~10^6^, were present. The overall time course and magnitude of the effects of H^+^ released by glia will thus depend critically on the concentration, spatial location, and variant of extracellular carbonic anhydrase present.

The pharmacological profile of ATP-initiated H^+^ release by glial cells in the intact nervous system is also an area worthy of future investigation that has the potential to be challenging and complicated. Our own experiments on astrocytes cultured from hippocampus and cortex strongly implicate activation of a P2Y receptor, since the ATP-initiated H^+^ flux could be largely abolished by thapsigargin, suggesting that release of calcium from intracellular stores is an essential step in the signal transduction pathway. The block of ATP-initiated H^+^ efflux by thapsigargin along with its elimination of ATP-elicited changes in intracellular calcium would appear to eliminate the possibility that calcium influx through a P2X receptor plays a significant role, since extracellular calcium was still present when ATP was applied in the presence of thapsigargin. Studies of ATP-elicited H^+^ efflux from retinal radial glia (Müller) cells strongly suggest activation of a PLC-dependent pathway leading to release of calcium stores, and similar studies should be done with the astrocyte cultures to solidify this portion of the transduction pathway. The specific subtype(s) of P2Y receptor initiating the signal transduction pathway is also a question worth pursuing, but also has the potential to be difficult to cleanly interpret. For example, data from retinal Müller cells of the tiger salamander suggest the possibility that six different subtypes of P2Y receptors are expressed by these cells (Reifel Saltzberg et al., 2003), and there is also the possibility of splice variants of P2Y receptors. Astrocytes in different parts of the nervous system are also likely to differ subtly from one another in their physiological and pharmacological profiles as neurons in different parts of the nervous system do (Batiuk et al., 2020; Borggrewe et al., 2020; Pestana et al., 2020), necessitating the careful determination of individual profiles of each subtype of astrocyte.

The experiments reported here demonstrate ATP-initiated H^+^ efflux from cells identified as astrocytes cultured without the presence of neurons. Several recently developed methods are likely to help shine light in future experiments designed to examine the role that H^+^ extrusion by glial cells plays in modulating neuronal activity in the intact nervous system. Molecular biological tools now allow selective activation of glial cell intracellular signaling cascade pathways using DREADDs (designer receptors exclusively activated by designer drugs), avoiding the potential problem of direct activation of ATP receptors also present on neurons in the intact nervous system (Xie et al., 2015; Bang et al., 2016; Losi et al., 2017; Yu et al., 2020). Such selective activation of glial cells coupled with the use of sensors such as CalipHluorin that allow measurement of changes in extracellular pH within intact individual synapses (Wang et al., 2014) could help to provide a much better understanding of the role that H^+^ efflux by glial cells plays in modulating neuronal activity. A key challenge in the interpretation of such studies will be in determining how similar the pattern of activation of alterations in intracellular calcium by DREADDs are compared to naturally occurring pathways both in magnitude and spatial pattern. Further experiments will also be needed for direct functional characterization of the effects of glial-related H^+^ on the inhibition of the release of neurotransmitters mediated by the block of calcium influx into neuronal axon terminals, as well as the extent to which such changes in glial-mediated alterations in extracellular acidity alter H^+^-dependent uptake of neurotransmitters. Our demonstration in this study of the direct release of H^+^ from astrocytes opens the door to a complex array of future studies needed to better understand the molecular mechanisms by which astrocytes may modulate neuronal activity.

## Data Availability Statement

The raw data supporting the conclusions of this article will be made available by the authors, without undue reservation.

## Ethics Statement

All animals were treated in accordance with the protocols and approved by the Animal Care Committee (ACC), the Institutional Animal Care and Use Committee (IACUC) of the University of Illinois at Chicago and the federal guidelines listed in the Public Health Service Policy on Humane Care and Use of Laboratory Animals.

## Author Contributions

JC, MK, and RM conceptualized the study, conducted experiments, analyzed resulting data, created figures, and discussed and wrote the manuscript. BT, WK, LK, and JL conducted experiments, analyzed resulting data, made suggestions for the direction of the study, and discussed and edited the manuscript. CG and MG contributed to the initial discussion about the design of the experiments using *in vitro* astrocyte cultures, provided cells for the study, helped guide preparation and culture of cells, and discussed and edited the manuscript. MK and RM wrote the grant applications that resulted in financial support for this work. All authors contributed to manuscript revision, read, and approved the submitted version.

## Conflict of Interest

LK declares his involvement with Spot Cells LLC. The remaining authors declare that the research was conducted in the absence of any commercial or financial relationships that could be construed as a potential conflict of interest.
